# Provision of menstrual products lowers school absenteeism in adolescent girls in coastal Kenya: findings from a quasi experimental study

**DOI:** 10.3389/frph.2026.1717803

**Published:** 2026-02-17

**Authors:** Lindah Sanyanda, Berrick Otieno, Kilian Mwadime, Mercedes Lu, Habibatou Traore, Cameron Kays, Lynette Kisaka, Wilhelm Hofstetter, Ishaq Makorani Y'Dhidha-a-Mjidho, Marianne Darwinkel

**Affiliations:** 1 Research Support Centre, North Coast Medical Training College, Kilifi, Kenya; 2 Department of Public Health, School of Health and Human Science, Pwani University, Kilifi, Kenya; 3 Environmental Law Alliance Worldwide, Eugene, OR, United States; 4 The Josef Korbel School of International Studies, Denver, CO, United States; 5 World Shoe Fund Non-Profit Social Enterprise, Charlotte, NC, United States; 6 University of Groningen, Groningen, Netherlands; 7 Department for Biomedical Research (DBMR), University of Bern, Bern, Switzerland; 8 Comundo, Luzern, Switzerland

**Keywords:** adolescent, menstrual health and hygiene, menstrual hygiene products, menstruation, reusable menstrual products, school attendance, Kenya, women's health

## Abstract

**Introduction:**

Despite global recognition of menstrual health as a public health priority, challenges in menstrual hygiene management (MHM) continue to affect adolescent girls' education in low- and middle-income countries. This study investigated knowledge, and perceptions on menstrual issues and the association of MHM product provision on school absenteeism among adolescent girls in Kilifi South Sub-County, in coastal Kenya.

**Methods:**

A mixed-methods design was implemented among 300 high school girls aged between 14 and 18 years in five secondary schools. A cross-sectional survey was conducted to assess MHM knowledge, menstrual practices, and school absenteeism. Focus group discussions were employed to explore perceptions regarding menstruation, product preferences, and challenges encountered during menses. Schools were randomly assigned to one of five study arms: a control group, non-reusable pads, reusable pads, menstrual cups, or all products with a choice option. Binary logistic regression with backward stepwise elimination method was used to determine factors associated with MHM knowledge. Kruskal–Wallis for inter-arm comparisons and Wilcoxon Signed Rank Test for within-arm comparisons were used to evaluate changes in absenteeism. Qualitative data were analyzed thematically using ATLAS.ti software.

**Results:**

In all schools, most of the participants demonstrated good MHM knowledge. Most of the participants use disposable sanitary pads, but financial constraints limit consistent access. Perceptions of menstruation were deeply influenced by cultural taboos and misinformation, contributing to stigma and influencing choice of menstrual product. Participants expressed a strong preference for disposable pads due to their ease of use and comfort, although financial constraints often necessitated the use of cheaper alternative products. Our intervention demonstrated significant reductions in menstruation-related absenteeism across all study groups. At baseline, the median menses-related school absenteeism score for all groups was 1 (IQR: 0–2), significantly decreasing to 0 (IQR: 0–1) (*p* < 0.001) at endline. Within study arms, significant declines in absenteeism were observed in the control group (median 1 to 0; *p* = 0.012), menstrual cup group (median 1 to 0; *p* = 0.041), disposable pads group (median 1 to 0; *p* < 0.001), and reusable cloth group (median 1 to 0; *p* < 0.001).

**Conclusion:**

The findings indicate that effective menstrual health management significantly improves school attendance among adolescent girls. Beyond product provision, addressing knowledge gaps, pain management, psychological support, and enhanced WASH infrastructure is essential. Increased awareness and open discussions about menstruation can also lead to positive outcomes. We recommend integrating comprehensive menstrual education into school curriculum, establishing sustainable menstrual product programs with complementary resources, and upgrading school WASH facilities to support female students.

## 1 Introduction

Menstruation is a natural physiological process that starts and continues in girls after puberty. The menstrual cycle typically spans around 28 days, though it varies (1). The onset of menstruation signals the readiness of a woman's body for potential pregnancy (2). In the absence of fertilization and subsequent implantation of a fertilized egg in the uterus, the body initiates a process where the thickened uterine lining, which had prepared itself to nourish a potential embryo, is no longer needed (3). This shedding of the uterine lining results in the release of blood and other materials, collectively referred to as menses, from the body through the vagina. The duration and intensity of menstrual flow can vary, and women often experience a range of physical and emotional symptoms (3).

Menstruation is associated with physical, medical, and psychosocial problems. Individuals may contend with premenstrual syndrome, menorrhagia (excessive menstrual bleeding), disrupted sleep patterns, bodily discomfort, and headaches (4). Menstrual medical disorders can arise from diverse factors, including issues related to ovulation, cycle length, and blood flow (5).Among adolescents, the risk of experiencing menstrual irregularities is elevated, attributable to hormonal imbalance, lifestyle choices, dietary patterns, and exercise habits (6). The psychosocial impact of menstruation goes beyond mood fluctuations, irritability, restlessness, and heightened stress levels (4) to include ridicule, abuse, physical harassment, and social isolation. For instance, individuals experiencing menstruation may face societal stigmatization and negative perceptions. Moreover, the menstrual cycle may increase psychiatric symptoms, with a documented heightened risk of psychosis, mania, depression, and alcohol use during the premenstrual and menstrual phases (7). Cumulatively, menstrual problems can translate into missed work and school days, exerting a notable influence on mental health (8) with stress particularly linked to dysmenorrhea and premenstrual symptoms (9). Furthermore, the painful menstrual cramps have been shown to negatively impact school attendance (4, 10).

Appropriate menstrual hygiene is crucial for managing flow-related problems during menstruation but is faced with challenges. Menstrual hygiene management (MHM) includes the use of sanitary materials to collect or absorb menstrual blood while also allowing for discreet replacement as necessary; following personal hygiene norms, such as the use of soap and water; as well as the availability of appropriate facilities for the correct disposal of menstrual waste (11). Appropriate menstrual hygiene management faces several challenges. Discriminatory attitudes and ideas pervade society, causing feelings of humiliation and contempt, which then lead to fear, teasing (12), social isolation (13), and restrictive rules in school settings (14). Adolescents especially from low and middle-income countries are often faced with the lack of proper amenities and resources, such as insufficient water supply, toilet facilities, and waste management systems (15) during menstruation, negatively impacting their school attendance (13) and their general well-being (16). Despite the availability of various menstrual products like tampons, disposable pads, reusable pads, and cups; personal preferences, cost of the products (17), cultural tolerance, and market availability dictates their utility (15).

Unmet menstrual hygiene needs are common and have adverse impacts on quality of life and school attendance. Some girls miss school during menses due to lack of sanitary pads (18). These unmet needs are further compounded by challenges in accessing menstrual hygiene-related information and social support (19). While some girls receive menstrual health information from their mothers, they generally perceive it as inadequate (16).

Several studies have demonstrated the effect of menstruation-related problems on school attendance. UNESCO estimates that one in ten girls in Sub-Saharan Africa misses school during her menstrual cycle (20) with some studies associating attendance with absence of gender-segregated toilets (16) or unlocked facilities at school (14). Cultural, and religious beliefs (such as those encouraging secrecy (21) surroundings menstruation) have also been linked to school attendance (16),underscoring the role played by some context-specific factors (15) and creating a need for local studies to quantify the effect of menstrual-related problems on school attendance (19).

Numerous studies have documented menstrual-related problems and their effect on school attendance (22), yet there remains a pressing need to address these challenges comprehensively (20). The Kenyan government has implemented various policies to address poor menstrual health and hygiene management, including repealing the value-added tax on sanitary pads in 2004, a commitment to provide free sanitary pads in public schools in 2010, and a 2017 amendment to the Education Act requiring the distribution of sanitary pads at schools (23). Additionally, international organisation such as the World Bank, UNICEF, WHO and United Nations have implemented various interventions to combat this problem in Kenya, however, there is limited data on MHM among adolescent girls, particularly in settings like the Kilifi County. Filling this gap is crucial to inform the Kenyan national and county governments under the Ministry of Health, Ministry of Education, and NGOs on refining intervention strategies-based effect of menstrual health on school attendance and the specific factors predisposing girls to adverse outcomes during menstruation.

This study aimed to explore and compare the MHM practices among school-going girls aged between 14 and 18 years in Kilifi south sub-county Kilifi County, with a specific focus (i) on the girls' menstrual knowledge, attitude and practices, (ii) perceptions towards different types of menstrual products and their utilization pattern, and (iii) the effect of menstruation issues on school attendance.

## 2 Methods

### 2.1 Area of study

The study was conducted in Kilifi South Sub-County, part of Kilifi County, between May and September 2024. Kilifi County is located on the coast, predominantly rural, and has a total population of 1,453,787 people (24). Kilifi is one of the poorest counties of Kenya with its poverty rate of 70.8% far exceeding the national poverty rate of 45.9% (24). Kilifi County is ranked among Kenya's regions with the lowest levels of literacy. The county faces numerous challenges, including a lack of potable water, inadequate sanitary facilities, limited access to healthcare services, prevalent health issues such as anemia (25) and malaria. Kilifi county has also a diverse range of religious affiliations, including Christians, Muslims, and traditionalists (25).

### 2.2 Study design

This study utilized a mixed-methods approach.

A cross-sectional study design was employed to assess knowledge, attitudes and practices of MHM through a structured questionnaire.

A qualitative research approach was used to explore perceptions surrounding MHM issues through focus group discussions. A quasi-experimental design was applied to examine the relationship between menstrual product access and school absenteeism through the same structured questionnaire which was submitted before and after intervention.

### 2.3 Description of the intervention

We provided three different menstrual products to different groups of girls to assess the relationship between menstrual product access and school absenteeism before and after this intervention as well as their perception of the different products.

The menstrual products provided differed between the different arms and were provided without regard to the type of product the girls had used before and were sufficient to support the girls during their menses for three months. The products included disposable pads, reusable pads, and menstrual cups. The study had five arms:

(i) Arm A: No intervention.
(ii) Arm B: Disposable pads (flora sanitary pads)
(iii) Arm C: Reusable pads (Washable cloths from “Days for girls”),
(iv) Arm D: Menstrual cups (silicone cups)
(v) Arm E: offered all three options and girls were free to choose one (28 participants chose reusable pads and 32 participants chose disposable pads, with none opting for the menstrual cup)

Prior to the distribution of products, girls received demonstrations on how to use and care for each type of menstrual product, along with instructional materials to assist them at home.

Immediately before implementation of the intervention, at the beginning of the school term, a structured questionnaire (the baseline) was completed by all participants to assess their knowledge, attitudes, practices, and school attendance related to menstrual health. After the 3 months, at the beginning of the next term, a similar structured questionnaire was again submitted (the endline) to assess any changes in school attendance during the intervention period.

### 2.4 Target population, study population, and sampling frame

Our target population for this study were adolescent, school going girls of poor and lower middle income families in Kilifi County who were considered at risk of missing access to sanitary pads and as a consequence might miss school. Our study population consisted of a total of 300 girls from 5 secondary, mixed, day schools in Kilifi-South.

The schools were purposely selected from a list of the 33 mixed secondary day schools in Kilifi South Sub-County. Out of these 33, 17 were dropped as they were private, and we expected the population in those schools to be of higher socio-economic standard. From the remaining 16, 5 schools were purposely selected based on spread in the Sub-County whereby they should be at least 5 km apart.

Each of the five schools was assigned to one of the five arms of the quasi-experimental study as described above.

Within each school, 60 girls who fulfilled the inclusion criteria were selected by random sampling from a sampling frame developed collaboratively with the school administration. Inclusion criteria included girls age 14 years to 18 years, enrollment duration of more than one academic term and having started menstruation. Girls who had significant physical or mental health concerns, were excluded.

From among the 300 randomly selected participants we again selected eight girls for participation in focus group discussions stratified for the years of study (two girls from each of the four years of secondary school).

### 2.5 Sample size and sampling techniques

For objective 1, the sample size was calculated using OpenEpi. The calculations were based on a population size of 1,800, with a hypothesized frequency of the outcome factor set at 80% ± 5%. The confidence limits were established at 5%, and the design effect for cluster surveys was set at 1. A sample size of 217 participants was required to achieve a 95% confidence level with a 5% margin of error in estimating MHM knowledge. To ensure robust statistical analysis and account for potential non-response or attrition, the sample size was increased by 30% to 300.

For the intervention, the sample size computation was informed by results from a similar study conducted in a rural setting that mirrored the current study (26). The calculations were based on a paired t-test to compare pre-intervention and post-intervention means. An effect size of 0.6 was assumed, representing the anticipated difference in mean school attendance in days between the baseline and end line. A significance level (α) of 0.05 was chosen to manage the risk of Type I error, while a targeted power (1-β) of 0.8 was set to achieve an 80% probability of correctly rejecting a false null hypothesis. Based on these parameters, a total sample size of 60 pairs of dependent observations was deemed sufficient.

### 2.6 Research instruments

The research instruments in this study included a structured questionnaire and focus group discussions. Through the structured questionnaires we gathered information on knowledge, attitudes, practices, and school attendance related to menstrual health and hygiene management. The focus group discussions were conducted to explore the girls' perceptions and utilization patterns of different types of menstrual products both reusable and non-reusable products.

#### 2.6.1 Pretest study

A pretest study was conducted to test the research instruments and procedures before the main data collection. The pretest study involved 10 high school girls from a different school that was not included in the main study. The pretest study assessed the clarity and comprehensibility of the questionnaires.

### 2.7 Data collection procedures

The data collection was done by trained data collectors in collaboration with the study investigators. The data collectors administered the structured questionnaire and conducted the focus group disussions, while LS and BO provided oversight and supervision. The quantitative data was collected using the KOBO Collect tool. The focus group discussions were captured using digital voice recorders. Participants’ consent was obtained before recording the discussions.

### 2.8 Data analysis

Quantitative data was analyzed using the statistical software STATA. Descriptive statistics was used to summarize the demographic characteristics, as well as the level of knowledge. Chi-square test was used to compare the distribution of study participants' knowledge on menstrual health and hygiene across various categories. Man–Whitney *U*-Test was used to compare the median age by knowledge status. Binary logistic regression was used to determine factors associated with knowledge on menstrual health and hygiene. In the bivariable analysis, we conducted independent models for each explanatory variable. We then performed backward stepwise elimination variable selection method in multivariable analysis. We started with a full model, including all candidate independent variables, and systematically removed the least significant variable at each step based on their *p*-values and stopped at *p*-value of 0.05. To determine the effect of menstrual products on school absenteeism, statistical comparisons were conducted at different stages of the study. First, a baseline comparison among the study arms was performed using the Kruskal–Wallis test to assess initial differences in absenteeism across groups. Similarly, the Kruskal–Wallis test was used to compare absenteeism among study arms at the endline, evaluating any differences that emerged after the intervention. Within-group comparisons were then conducted using the Wilcoxon Signed Rank Test to assess changes in absenteeism from baseline to endline within each study arm. Finally, an overall comparison between baseline and endline absenteeism across all participants was carried out using the Wilcoxon Signed Rank Test to determine the general effect of menstrual product access on absenteeism.

The qualitative data were analyzed using thematic analysis. First, all recordings were transcribed verbatim. The transcripts were then read multiple times for familiarization, and initial codes were generated using an inductive approach. These codes were systematically categorized into broader themes that captured key patterns and insights from the data. To enhance credibility, coding was conducted independently by two researchers (LS and BO), and discrepancies were resolved through discussion and consensus. The identified themes were further examined for relationships and meanings relevant to the study objectives. ATLAS.ti software was used to organize and manage the data.

### 2.9 Logistical and ethical considerations

Informed consent was secured from a senior teacher, typically the principal or their deputy, along with assent from participants. The process involved explaining the study, including potential discomforts and benefits, to the individuals and the teacher in an appropriate language (Kiswahili or English). The school administration, including the school principals, provided consent on behalf of the parents and guardians. To maintain the confidentiality of the information gathered, the electronic devices used for data collection in the field were password-protected, with access limited to the user and the supervisor. Daily, the data were uploaded to a central database, which was also secured by passwords known only to the principal investigator. To ensure anonymity, all participants were assigned a study code number, which was the only identifier saved in the main database. A separate file linking personal identifiers to code numbers was stored separately in the principal investigator's computer. For the participants to have an equal benefit of participation, the control group was also given menstrual products after the intervention.

## 3 Results

Table 1 presents the demographic and menstrual health management (MHM) related characteristics of the study participants. 300 high school girls took part, with a median age of 17 years (IQR = 16–18). Most participants were Christians, 249 (83.0%). Of the 300 participants, 84(28.0%) indicated receiving information on MHM primarily from their parents or guardians. On the type of menstrual flow, most participants, 201(67.0%) reported having a normal flow. Additionally, 160 participants (53.3%) were likely to discuss menstrual issues with teachers. Most participants expressed a willingness to discuss MHM issues with their parents (86.0%), friend (64.0%) and healthcare workers (75.7%) More than half of the participants reported having missed at least a school day.

**Table 1:** Demographic and menstrual health management (MHM) related participants characteristics.

Participants characteristics			Frequency *N* (%)
Age; median (IQR)			17 (16–18)
Religion		Christians	249 (83.0)
		Muslim	51 (17.0)
Source of information		Parents/guardians	84 (28.0)
		Others also	216 (72.0)
Amount of flow		Heavy	61 (20.3)
		Light	38 (12.7)
		Normal	201 (67)
Discussing menstrual issues	Teachers	Unlikely	140 (46.7)
		Likely	160 (53.3)
	Parents	Unlikely	42 (14)
		Likely	258 (86)
	Friends	Unlikely	108 (36)
		Likely	192 (64)
	Healthcare workers	Unlikely	73 (24.3)
		Likely	227 (75.7)
Absenteeism		No	149 (49.7)
		Yes	151 (50.3)

### 3.1 Menstrual health knowledge stratified by school

**Figure 1:**
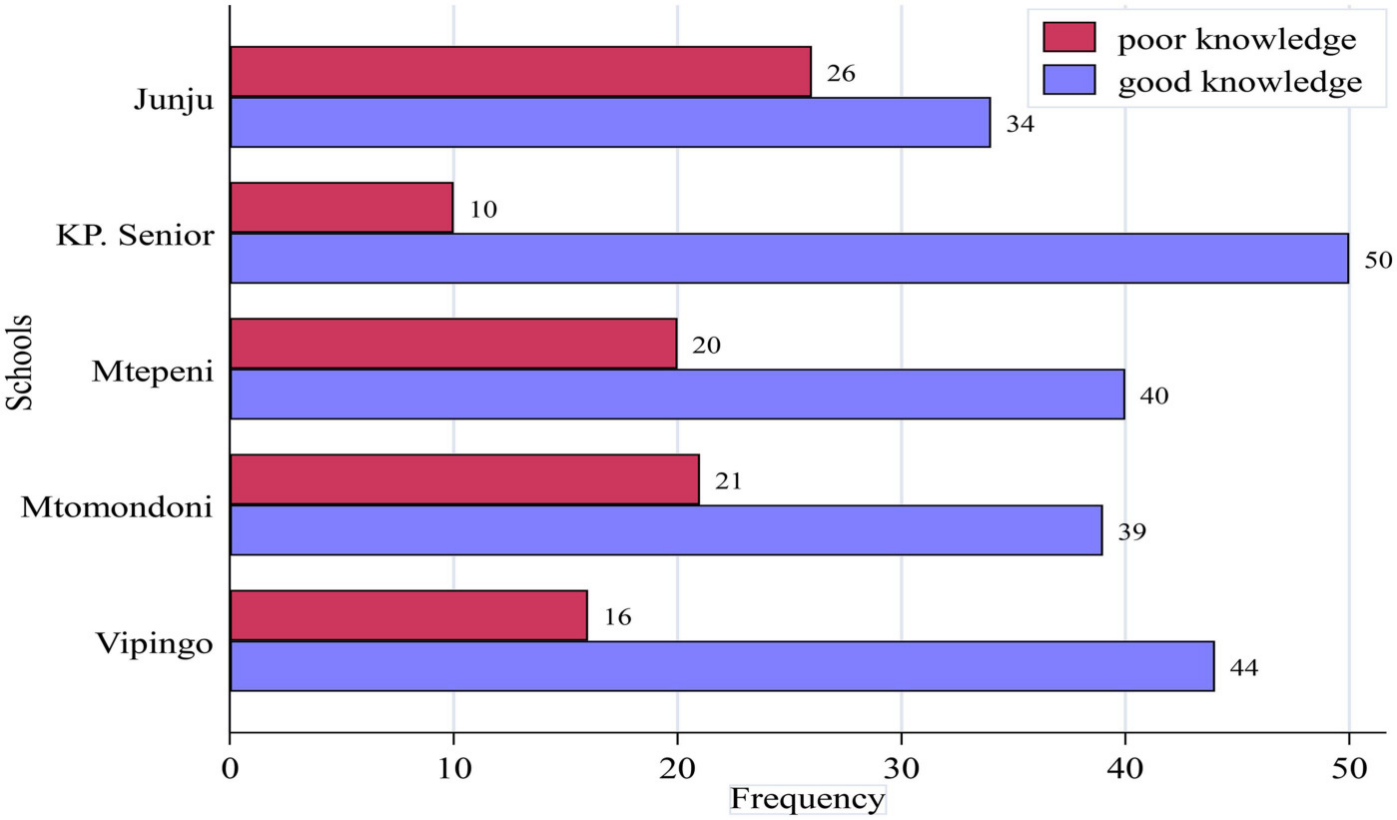
Menstrual health knowledge stratified by schools.

The bar graph (Figure 1) presents a finding of menstrual health knowledge among participants from Arm A, Arm B, Arm C, Arm D and Arm E secondary schools. The data categorizes participants knowledge into poor knowledge (red bars) and good knowledge (blue bars), with the frequency of respondents shown for each category. All schools demonstrated more students with good menstrual health knowledge than poor knowledge, though the proportions varied significantly. Arm D exhibited the best performance, with 50 (83.3%) participants showing good knowledge compared to just 10 with poor knowledge. Arm C with 44 (73.3%) participants demonstrating good knowledge. In Arm E, 40 (66.7%) participants had good knowledge while Arm A had 39 (65%) participants with good knowledge, Arm B showed the lowest performance with only 34 (56.7%) participants demonstrating good knowledge. This places Arm B at the bottom of the comparative ranking, though it still maintains more students with good knowledge than poor knowledge.

### 3.2 Knowledge on menstrual issues stratified by participants characteristics

There were no significant differences in MHM knowledge levels based on age (*p* = 0.615) or religion (*p* = 0.110). However, MHM knowledge levels differed significantly among participants from different schools (*p* = 0.025) (Table 2). In terms of the source of information, no significant differences were found in MHM knowledge between those who reported receiving information from parents or guardians vs. other sources (*p* = 0.586). There were no significant differences in MHM knowledge levels based on the flow amount (*p* = 0.571). The likelihood of discussing menstrual health issues with teachers (*p* = 0.250), parents (*p* = 0.994), and friends (*p* = 0.7) did not significantly differ between those with poor and good knowledge. However, a higher proportion of participants with good knowledge were likely to discuss menstrual issues with healthcare workers compared to those with poor knowledge, with this difference approaching statistical significance (*p* = 0.064).

**Table 2:** Knowledge on menstrual issues stratified by participants characteristics.

Participants characteristics			Knowledge		
			Poor *N* (%)	Good *N* (%)	*P*-value
Age; median (IQR)			17 (16–18)	17 (16–18)	0.615^a^
Religion		Christians	82 (88.2)	167 (80.7)	0.110
		Muslim	11 (11.8)	40 (19.3)	
School		Junju	26 (27.96)	34 (16.4)	**0**.**025**
		\KP. Senior	10 (10.8)	50 (24.2)	
		Mtepeni	20 (21.5)	40 (19.3)	
		Mtomondoni	21 (22.6)	39 (18.8)	
		Vipingo	16 (17.2)	44 (21.3)	
Source of Information		Parents/guardians	28 (30.1)	56 (27.1)	0.586
		Others also	65 (69.9)	151 (73)	
Amount of flow		Heavy	19 (20.4)	42 (20.3)	0.571
		Light	9 (9.7)	29 (14)	
		Normal	65 (69.9)	136 (65.7)	
Discussing menstral issues	Teachers	Unlikely	48 (51.6)	92 (44.4)	0.250
		Likely	45 (48.4)	115 (55.6)	
	Parents	Unlikely	13 (14)	29 (14)	0.994
		Likely	80 (86)	178 (86)	
	Friends	Unlikely	32 (34.4)	76 (36.7)	0.700
		Likely	61 (65.6)	131 (63.3)	
	Healthcare workers	Unlikely	29 (31.2)	44 (21.3)	0.064
		Likely	64 (68.8)	163 (78.7)	
Absenteism		No	34 (36.6)	115 (55.6)	**0**.**002**
		Yes	59 (63.4)	92 (44.4)	

All *p* values are from persons Chi Square Test unless specified.

^a^ *p* value from Man–Whitney *U*-Test.

### 3.3 Factors associated with knowledge on menstrual issues

Bivariable logistic regression identified significant associations between knowledge levels and specific variables (Table 3). School emerged as a strong predictor of menstrual health knowledge. Students from KP Senior demonstrated significantly higher odds of good knowledge compared to those from Junju (OR = 3.8, 95% CI: 1.6–8.9, *p* = 0.002). Absenteeism was negatively associated with knowledge levels, with students reporting absence from school showing lower odds of possessing good knowledge (OR = 0.5, 95% CI: 0.3–0.8, *p* = 0.006). The likelihood of discussing menstrual issues with healthcare workers showed a positive trend toward better knowledge, though this association approached but did not reach statistical significance (OR = 1.7, 95% CI: 0.9–3.1, *p* = 0.065). Age (*p* = 0.615), source of information (*p* = 0.586), and menstrual flow amount (*p* = 0.242) showed no significant association with knowledge levels in the bivariable analysis. After adjusting for potential confounders school attendance remained a significant independent predictor of menstrual health knowledge (Table 3). Students from KP Senior maintained higher odds of good knowledge compared to Junju students (adjusted OR = 2.4, 95% CI: 1.2–5.1, *p* = 0.019). Similarly, absenteeism remained negatively associated with knowledge levels (adjusted OR = 0.5, 95% CI: 0.3–0.8, *p* = 0.006).

**Table 3:** Factors associated with knowledge on menstrual issues.

Factors			Bivariable analysis		Multivariable analysis	
			Odds Ratio OR (95% CI)	*P*-value	Odds ratio OR (95% CI)	*P*-value
Age; median (IQR)			1 (0.8–1.2)	0.694	^¶^	
Religion		Christians	1 (Reference)	.		
		Muslim	1.8 (0.9–3.7)	0.113	^¶^	
School		Junju	1 (Reference)	.	1 (Reference)	
		KP. Senior	3.8 (1.6–8.9)	0.002	2.4 (1.2–5.1)	0.019
		Mtepeni	1.5 (0.7–3.2)	0.261	^¶^	
		Mtomondoni	1.4 (0.7–3)	0.35	^¶^	
		Vipingo	2.1 (1–4.5)	0.057	^¶^	
Source of Information		Parents/guardians	1 (Reference)	.	^¶^	
		Others also	1.2 (0.7–2)	0.586	^¶^	
Amount of flow		Heavy	1 (Reference)	.	^¶^	
		Light	1.5 (0.6–3.7)	0.424	^¶^	
		Normal	0.9 (0.5–1.8)	0.861	^¶^	
Discussing menstral issues	Teachers	Unlikely	1 (Reference)		^¶^	
		Likely	1.3 (0.8–2.2)	0.25	^¶^	
	Parents	Unlikely	1 (Reference)	.	^¶^	
		Likely	1 (0.5–2)	0.994	^¶^	
	Friends	Unlikely	1 (Reference)	.	^¶^	
		Likely	0.9 (0.5–1.5)	0.7	^¶^	
	Healthcare workers	Unlikely	1 (−)		^¶^	
		Likely	1.7 (1–2.9)	0.065	^¶^	
Absenteeism		No	1 (Reference)	**0**.**003**	1 (Reference)	**0**.**006**
		Yes	0.5 (0.3–0.8)		0.5 (0.3–0.8)	

^¶^ Variables eliminated from the model.

### 3.4 Perceptions menstrual issues and preferences of menstrual products

#### 3.4.1 Understanding of menstruation and menstrual products

The study revealed varied levels of understanding of menstruation among participants. While some respondents demonstrated a basic understanding, describing menstruation as the discharge of blood, others recognized it as a process linked to ovulation, occurring approximately every 28 days.

For example, one participant noted, “*The process of discharging blood in females*” (Respondent 1, Mtomondoni), while another stated, “*The losing of an ovum after every 28 days*” (Respondent 3, KP Senior). Despite this understanding, misconceptions were prevalent, particularly regarding the age of onset, with some participants believing menstruation could begin as early as 7 years. General awareness of the menstrual cycle's typical duration of 28 days was observed, though comprehension varied. Some participants acknowledged that cycles could be regular or irregular, while others demonstrated limited knowledge of the physiological aspects. One participant noted, “*After 28 days*” (Respondent 3, Mtepeni), while another recognized variability, stating, “*Sometimes it can skip, coming either earlier or later*” (Respondent 3, Mtomondoni).

Participants were generally familiar with menstrual hygiene products, with disposable pads being the most widely recognized and commonly used. As one participant affirmed, “*Yes, everyone has used that*” (Respondent 10, Mtomondoni). In contrast, tampons were less commonly recognized, with some participants reporting that they had heard of them but had never seen or used them, stating, “*We have heard but never seen it*”. Similarly, menstrual cups and reusable pads were acknowledged but had not been used by any participant, with one noting, “*We haven't even seen them in shops*” (Respondent 6, Junju).

#### 3.4.2 Preferences and selection of menstrual products

Disposable pads emerged as the most preferred menstrual product due to their convenience, comfort, and accessibility. One participant emphasized, “*Yes, disposable pads have advantages*” (Respondent 6, Vipingo), while another cited ease of disposal as a key factor: “*Because I can only use it once and then dispose of it*” (Respondent 6, Vipingo). Cost was a major determinant in product selection, with affordability concerns influencing purchasing decisions. As one participant highlighted, “*It's expensive*” (Respondent 3, Vipingo). Reusable pads were considered a cost-effective alternative, as one participant explained, “*You can buy them once and use them over and over again*” (Respondent 4, Mtomondoni). Another participant added, “*[They] help when you don’t have money, you can wash it, dry it, and reuse it*” (Respondent 7, Mtepeni). While reusable pads such as cloth were perceived as economical, they were also associated with practical challenges, including washing, drying, and storage. Some participants expressed concerns about hygiene and logistical constraints, particularly in school settings. As one participant noted, “*If you are having a heavy flow, and change it while at school, where will you keep the filled cloth?*” (Respondent 2, Junju). Others found the process of washing and drying reusable pads inconvenient and culturally sensitive, stating, “*It's not hygienic*”, and “*It's hard to clean. When you wash them, it needs sunlight*” (Respondent 3, Junju). Menstrual cups were met with skepticism and cultural concerns, with some participants associating them with virginity loss, difficulty inserting them, lack of exposure to menstrual cups, as one respondent asserted, “*It can break your virginity if not placed well*” (Respondent 2, KP Senior).

#### 3.4.3 Barriers to menstrual hygiene management

Financial barrier was a primary constraint, limiting access to menstrual products. As one respondent explained, “*If you don't have money, you can’t find one*” (Respondent 2, Vipingo). Stigma surrounding menstruation also affected participants' experiences, with many feeling uncomfortable discussing the topic, particularly in the presence of male family members, teachers, or peers. Some participants reported that they could not ask their fathers for menstrual products, instead seeking support from their mothers. One participant remarked, “*We don't ask from our dads, we ask from the mothers*” (Respondent 5, Vipingo), while another added, “*Our fathers are traditional, they don't want to hear anything*” (Respondent 8, Mtomondoni). Cultural practices and misconceptions contributed to misinformation and stigma. Some respondents recalled traditional practices that influenced their menstrual management, with one participant sharing, “*They ask you to dig a hole in the sand and you have to sit there*” (Respondent 3, Mtomondoni). Feelings of embarrassment also led some participants to avoid purchasing menstrual products themselves, opting instead to send someone else to buy them. As one participant noted, “*I send someone to buy the pads instead of buying them myself*” (Respondent 5, Mtepeni). The school environment further exacerbated these challenges, with inadequate facilities for menstrual hygiene management. Many participants reported a lack of private and functional washrooms, insufficient water and soap, and no designated times for product changes. One participant stated, “*There is no water at school*” (Respondent 3, Junju). Fear of stigma from peers and teachers, coupled with rigid school schedules, further hindered effective menstrual hygiene management.

#### 3.4.4 Menses -related school absenteeism

Table 4 presents a comparison of menses-related school absenteeism scores between baseline and endline, as well as within the study arms. At baseline, the median menses-related school absenteeism score for all participants was 1, with an interquartile range (IQR) of 0 to 2. This indicates that, at the beginning of the study, half of the participants reported rarely missing school due to menstruation, with interquartile range from 0 (never) to 2 (occasionally) days missed. By the endline assessment, the median absenteeism score for all participants had decreased to 0 (IQR: 0–1). This shift demonstrates a general reduction in menses-related school absenteeism across the study population. This overall change, difference between baseline and endline, was statistically significant (*p*-value = 0.000). Further analysis examined the changes within each study arm. In the control group, the median absenteeism score decreased from 1 (IQR: 0–1) at baseline to 0 (IQR: 0–1) at endline. This reduction was statistically significant (*p*-value = 0.012). Similarly, the menstrual cup group also showed a significant decrease in the median score from 1 (IQR: 0–1) at baseline to 0 (IQR: 0–1) at endline (*p*-value = 0.0407). The disposable pads group showed a statistically significant decrease from 1 (IQR: 0–1) at baseline to 0 (IQR: 0–0) at endline (*p*-value = 0.0004). The reusable cloth group also exhibited a statistically significant decrease, from a median of 1 (IQR: 0–2) at baseline to 0 (IQR: 0–0) at endline (*p*-value = 0.0001).

**Table 4:** Comparison of the distribution of menses-related school absenteeism score between baseline and endline and within study arms.

Categories	Menses-related school absenteeism score						*P*-value^c^	*P*-value^d^
	Baseline			Endline				
	Median (IQR)	Rank sum	*P*-value^a^	Median (IQR	Rank sum	*P*-value^b^		
All	1 (0–2)			0 (0–1)				**0** **.** **0000**
Control	1 (0–1)	9,251.00	0.4000	0 (0–1)	9,490.50	0.3085	**0**.**0120**	
Menstrual Cup	1 (0–1)	8,503.50		0 (0–1)	9,363.00		**0**.**0407**	
All options	1 (0–2)	8,536.50		0 (0–1)	8,827.00		0.0550	
Disposable pads	1 (0–1)	8,115.50		0 (0–0)	7,999.00		**0**.**0004**	
Reusable Cloth	1 (0–2)	9,846.50		0 (0–0)	8,573.50		**0**.**0001**	

^a^ Comparison among study arms at baseline (*p*-value from Kruskal Wallis Test).

^b^ Comparison among study arms at endline (*p*-value from Kruskal Wallis Test).

^c^ Comparison within study arms at baseline and endline (*p*-value from Wilcoxon Signed Rank Test).

^d^ Overall comparison between baseline and end-line (*p*-value from Wilcoxon Signed Rank Test).

## 4 Discussion

This study assessed 300 girls across five public secondary schools in Kilifi South Sub County, Kenya, revealing gaps in menstrual health management (MHM) and its effect on school attendance. The findings highlight the interconnection between knowledge, beliefs, and resource availability that significantly influences girls' menstrual experiences. Majority participants exhibited good knowledge on menstrual issues; however, some only learnt about menstruation upon reaching menarche. Only 28% of participants reported receiving menstrual education from parents. Previous studies (13) have reported that inadequate education on menstruation contributes to fear (14) and confusion among girls (27). Based on our study, indeed it confirms that also in the area of Kilifi South, there is need for enhanced menstrual health education, both at home and in school.

Our baseline findings report that most of the participants were using disposable pads, with participants noting these products as easy to use and comfortable. However, financial challenges report by many participants highlight that affordability remains a significant barrier. For the menstrual cups and reusable pads, participants acknowledged that they have heard of them but none of them had used them This aligns with evidence suggesting that economic limitations restrict access to essential menstrual products, often leading girls to resort to less effective alternatives such as cloths (8). Access to reliable menstrual products was shown to significantly influence girls' confidence (28) and ability to manage menstruation effectively (29). Programs by the government and nongovernmental organizations should focus on availing acceptable products to address period poverty.

Some of the barriers to effective MHM identified in this study included financial constraints, stigma, cultural beliefs, and inadequate water, sanitation, and hygiene (WASH) facilities in schools, all of which disrupt girls' ability to manage menstruation. Many schools were found to lack private and clean toilets equipped with water and soap, which are essential for effective menstrual health management. These findings corroborate other research (16) indicating that inadequate WASH facilities directly correlate with increased school absenteeism (30), as girls often choose to stay home to avoid embarrassment (31). Many girls expressed fear of ridicule from peers, particularly regarding staining their uniforms while at school, which caused discomfort and distraction in the classroom. This aligns with other studies that have reported that fear, stigma (13), and psychological distress significantly contribute to school absenteeism during menstruation (32).

There were improvements in school absenteeism linked to menstruation following interventions. Across all study groups, a significant reduction in absenteeism was observed, with the most pronounced effects seen in participants provided with disposable pads and reusable pads. This aligns with existing evidence that reliable menstrual products can alleviate practical barriers to school attendance, such as fear of leakage or discomfort, thereby fostering continuity in education (13). The control group's improvement, though unexpected, may reflect heightened awareness or behavioral shifts stemming from study participation, a phenomenon often attributed to the Hawthorne effect, wherein individuals modify their behavior due to the awareness of being observed (33). Absenteeism also decreased in the control arm, with possible confounding factors including the participants' awareness that data collectors would return to their school in three months, even though they were unaware of the specific evaluation criteria which may have triggered behavioral change. Lastly, the repeated administration of the same questionnaire from baseline to endline may have introduced a questionnaire effect, influencing the reduction in school absenteeism due to participants' familiarity with the questions. In contrast, the endline results reflect attendance during the second term, which is typically when students are more settled, as it is the longest of the three terms.These findings emphasize the value of even non-material interventions, such as sensitization or open dialogue around menstruation, in addressing absenteeism.

### 4.1 Limitations

While our study provides valuable insights into menstrual health management among adolescent girls in Kilifi County, several limitations warrant consideration. First, the quasi-experimental design without full randomization may introduce selection biases that could affect the validity of our causal claims regarding intervention effects. Second, the three-month intervention period, while sufficient to observe immediate effects, may be inadequate to evaluate long-term sustainability of behavior changes or product acceptability, particularly for unfamiliar products like menstrual cups that typically require longer adaptation periods.

Our reliance on self-reported data for both menstrual experiences and school attendance introduces potential recall bias and social desirability effects, especially given the sensitivity of the topic. The observed improvements in the control group suggest possible Hawthorne effects that complicate the interpretation of intervention impacts. Our focus on in-school adolescents excludes out-of-school girls who may face more severe menstrual health challenges, limiting the generalizability of our findings to the broader adolescent population. Additionaly we could not verify the utilization of the selected products by participants during the endline survey, this constraint has reduced our ability to draw firm conclusions regarding the actual usage of the provided products among the participants. Finally, while we documented the association between menstrual product access and absenteeism, our study design cannot fully disentangle the relative contributions of product availability vs. other concurrent factors such as education, psychological support, and improvements in school facilities.

## 5 Conclusion and recommendations

This study demonstrates that menstrual health management (MHM) significantly impacts school attendance among adolescent girls, with improvements observed following interventions. Our findings reveal that while menstrual product provision is important, a comprehensive approach addressing knowledge gaps, pain management, psychological support, and WASH infrastructure is essential for meaningful impact. The unexpected improvement in the control group highlights how even increased awareness and normalization of menstruation discussions may yield positive outcomes.

Based on these findings, we recommend several evidence-based strategies for policy implementation. First, educational institutions should integrate comprehensive menstrual health education into school curricula, targeting both girls and boys to reduce stigma and misinformation. This education should begin before menarche and involve parents to strengthen home-based support systems. Government and non-governmental stakeholders should establish sustainable programs for menstrual product provision, offering a range of options (disposable pads, reusable products) to accommodate different preferences and sustainability concerns. These initiatives should include complementary resources such as menstrual health products and pain management education. Investments in school infrastructure must prioritize female-friendly WASH facilities, including private changing areas, clean water access, and proper disposal systems for menstrual waste. Based on various other findings not presented in this article from our study, dysmenorrhea was a problem that should also be addressed. Therefore, healthcare systems should integrate menstrual health into primary care services, with particular attention to dysmenorrhea management and psychological support. Finally, future research should explore the long-term impacts of comprehensive MHM interventions, investigate cost-effective scaling strategies, and evaluate the effectiveness of technology-based educational approaches in resource-limited settings.

## Data Availability

The original contributions presented in the study are included in the article/Supplementary Material, further inquiries can be directed to the corresponding author.
